# PDE-Mediated Cyclic Nucleotide Compartmentation in Vascular Smooth Muscle Cells: From Basic to a Clinical Perspective

**DOI:** 10.3390/jcdd9010004

**Published:** 2021-12-22

**Authors:** Margarida Lorigo, Nelson Oliveira, Elisa Cairrao

**Affiliations:** 1 Health Sciences Research Centre (CICS-UBI), University of Beira Interior, 6200-506 Covilha, Portugal; margarida.lorigo@gmail.com; 2 Department of Medical Sciences, Faculty of Health Sciences (FCS-UBI), University of Beira Interior, 6200-506 Covilha, Portugal; 3 UDI-IPG, Research Unit for Inland Development, Department of Social Sciences and Communication, Polytechnic Institute of Guarda, 6300-654 Guarda, Portugal; nelsonoliveira@ipg.pt

**Keywords:** compartmentation, cAMP, cGMP, vascular smooth muscle cells, cardiovascular diseases

## Abstract

Cardiovascular diseases are important causes of mortality and morbidity worldwide. Vascular smooth muscle cells (SMCs) are major components of blood vessels and are involved in physiologic and pathophysiologic conditions. In healthy vessels, vascular SMCs contribute to vasotone and regulate blood flow by cyclic nucleotide intracellular pathways. However, vascular SMCs lose their contractile phenotype under pathological conditions and alter contractility or signalling mechanisms, including cyclic nucleotide compartmentation. In the present review, we focus on compartmentalized signaling of cyclic nucleotides in vascular smooth muscle. A deeper understanding of these mechanisms clarifies the most relevant axes for the regulation of vascular tone. Furthermore, this allows the detection of possible changes associated with pathological processes, which may be of help for the discovery of novel drugs.

## 1. Introduction

Cardiovascular diseases (CVDs) are important causes of mortality and morbidity worldwide [1,2]. Vascular smooth muscle cells (SMCs) are the main components of blood vessels located in the middle layer (tunica media) of the vessel wall [3,4]. These cells have an important role both in physiological and pathophysiological conditions [3,5,6]. In healthy vessels, vascular SMCs contribute to vasotone and for the regulation of blood flow by cyclic nucleotide intracellular pathways [5,7]. The cyclic nucleotides, cyclic adenosine-3′,5′-monophosphate (cAMP) and cyclic guanosine-3′,5′-monophosphate (cGMP), are important second messengers that regulate a series of functions at the cellular level [5]. The synthesis of cAMP is catalysed by adenylyl cyclases (AC) and regulated by G-protein-coupled receptors (GPCR), while cGMP is generated by soluble and particulate guanylyl cyclases (GC). While soluble GC (sGC) are activated directly by nitric oxide (NO), particulate GC (pGC) are activated by natriuretic peptide receptors [8,9,10]. On the other hand, the amplitude and duration of cyclic nucleotide responses are controlled by their degradation by cyclic nucleotide phosphodiesterases (PDEs) [5,11]. The onset of certain pathological conditions, such as atherosclerosis or hypertension, could trigger a process of vascular SMCs remodelling, known as phenotypic switching, in which they lose their contractile phenotype and alter contractility or their signalling mechanisms [12,13]. Furthermore, this phenotypic switching may also occur in response to changes in the local environment, demand, pregnancy, bodybuilding, vascular development, in which the SMCs exhibit a high rate of proliferation and migration, i.e., they exhibit a synthetic phenotype [14,15,16]. Moreover, SMCs may undergo changes in contractility or signalling mechanisms, including the compartmentation of cyclic nucleotides [17]. This process involves several mechanisms by which multiple spatially segregated cAMP and cGMP signalling pathways exert different or even opposite functional effects on distinct subcellular microdomains of the same cell [5,18,19]. Therefore, compartmentation is a crucial aspect of intracellular signalling, which coordinates the precise signal propagation and the specificity of signalling outcomes [20].

In the present review, we focus on compartmentalized signaling of cyclic nucleotides in vascular smooth muscle. We provide a detail of the components involved in the compartmentalized signaling of cyclic nucleotides, explain the compartmentation of each (how they interact in signalling), and critically discuss from basic to clinical perspective how compartmentation may be a potential therapeutic approach for cardiovascular diseases.

## 2. Vascular Smooth Muscle

The main function of vascular smooth muscle is to regulate vascular tone. In this process, the signalling cascades regulate contraction and relaxation [3] in response to various hormonal and hemodynamic stimuli. Therefore, SMCs are the cells responsible for the contractile property of blood vessels, which are involved in the regulation of blood pressure and blood flow distribution [12,21].

The SMC contraction mechanism is triggered by an increase of cytosolic Ca^2+^ concentration via two pathways: (1) a transient and rapid increase through Ca^2+^-release from the SR and/or (2) sustained extracellular Ca^2+^ influx through Ca^2+^ channels [22,23]. In this process, Ca^2+^ binds to calmodulin (CaM), which induces a CaM conformational change and the formation of the Ca^2+^/CaM complex. In turn, this complex activates the myosin light chain kinase (MLCK) that phosphorylates one of the myosin light chains activating the cross-bridge cycle resulting in SMC contraction [5,24,25].

**Figure 1:**
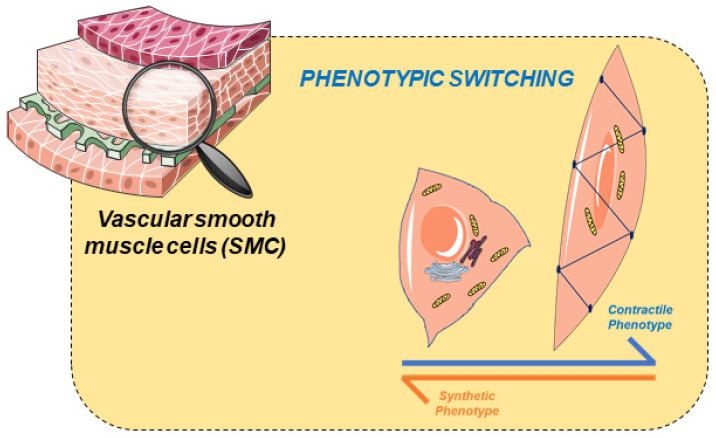
Schematic representation of the phenotypic switching in vascular smooth muscle cells.

SMCs are highly specialized since they express a variety of contractile proteins, specific types of ionic channels, enzymes, and membrane receptors, allowing them to regulate contractile function [26,27]. The different vascular functions of these cells result from a diversity of phenotypes (ranging from contractile to synthetic). These two phenotypes, which represent the opposite ends of the SMC phenotypic spectrum, are characterized by well-defined structural features with respect to morphology, proliferation, and differential protein expression [28,29]. Thus, vascular SMCs are plastic [30], and respond to adverse conditions by modulating their phenotype through a process called “phenotypic modulation” (Figure 1). In this process, SMCs have the potential to switch between phenotypes, either towards a more contractile, or towards a more synthetic phenotype. This phenotypic modulation has been associated with vascular injury as vascular tone regulation is reduced [31,32], and the cells become synthetic cells. The underlying molecular mechanisms are still unclear. 

Regarding structural features, cells displaying the contractile phenotype have an elongated spindle shape. When cells change to a synthetic phenotype, they remain less elongated and acquire a parallelepiped (also referred to as epithelioid or rhomboid) morphology [31] similar to endothelial cells [33]. Contractile cells decrease their protein expression markers and increase their proliferation, migration, and production, i.e., becoming synthetic cells [3,34]. The diversity of these cells does not compromise the performance of the arteries. However, it confers the essential flexibility to respond to different physiological or pathological situations [28]. However, some studies suggest that vascular injury triggers the phenotypic modulation in SMCs [35], but the molecular mechanisms that can lead to this process are still unclear. In summary, there is strong evidence that phenotypic alteration in SMCs plays a significant role in atherosclerosis, hypertension, and preeclampsia. 

## 3. Vascular Cyclic Nucleotides

The cyclic nucleotides cAMP and cGMP are essential modulators of vascular function. Therefore, knowledge about cyclic nucleotides signalling is crucial to understand the modulatory effects of these nucleotides on vascular smooth muscle regulation, and these cyclic nucleotides are the main intracellular messengers associated with SMC vasodilation [5].

The intracellular levels of cyclic nucleotides are regulated by the enzymes that synthesize (ACs), and that degrade (PDEs) [5]. The molecular mechanisms involved in the generation of cAMP and cGMP have been described [8,36]. Briefly, cAMP synthesis occurs after AC stimulation, and is usually stimulated by GPCR linked to stimulatory G (Gs) proteins. After stimulation of AC, adenosine triphosphate (ATP) is dephosphorylated, and cAMP and pyrophosphate (PPi) are produced [8]. Regarding cGMP synthesis, this occurs through the stimulation of pGC or sGC. The pGC can be stimulated by natriuretic peptides (NP), whereas sGC is stimulated by NO or NO donors. After stimulation of GC, guanosine triphosphate (GTP) is dephosphorylated, and cGMP and PPi are produced [8].

The ACs are 12 transmembrane proteins that catalyses the conversion of ATP to cAMP in the presence of Mg^2+^ [37,38,39,40]. These cyclases are normally activated by an external signal (neurotransmitter, hormone, or drug) that binds to GPCRs [5,38]. The ACs are currently divided into six unrelated classes, five of which (classes I, II, IV, V, VI) have not been studied in detail, mostly because they are limited to a narrow range of prokaryotic species. Class III is numerically the largest, structurally and functionally most diverse, and pharmacologically most relevant; it is also the only one present in animals [41]. In this class there are nine transmembrane AC (mAC) isoforms (AC1–9) and a soluble AC (sAC, also designated as AC10) [38,39,40,41,42]. All membrane isoforms have an identical structure, being single peptide chains that consists of an N-terminal cytosolic domain of varying length, two membrane-spanning domains (TM1, TM2), each with six transmembrane α-helices, and two cytosolic domains C1 and C2 subdivided into catalytic (C1a, C2a) and regulatory (C1b, C2b) subdomains [39,43]. Isoforms with the catalytic domain C1a are regulated by Gi, while Gs regulate those with the catalytic domain C2a. Gi is thought to cause a rotation of the C1a regions in the opposite direction to Gs, and it is this type of rotation that decreases of the enzymatic activity of the AC [40,44].

Overall, AC can be regulated in different ways [37,39,40,45] and depending on the properties and relative levels of isoforms expressed in each tissue or cell type, the extracellular signals received through the receptors can be differentially integrated [45]. Usually, all membrane isoforms are stimulated by the α-subunit of the Gs protein [39,40,43], and some are inhibited by Gi protein [40]. In addition, the plant diterpene forskolin (FSK) from Coleus forskohlii (an ancient Indian folk medicine) has also been identified as an AC activator. FSK has hypotensive effects [44,46], and is an essential experimental tool for the study of the involvement of AC in (patho)physiologic processes [39]. In vascular SMCs, the AC2, AC3, AC5, AC6, and AC8 have been described, but the quiescent vascular SMCs expressed mainly the AC3, AC5, and AC6 and the dedifferentiated cultured vascular SMCs express mainly AC2 and AC8 [7]. These AC isoforms are also regulated by phosphorylation through the activation of protein kinases such as Ca^2+^/calmodulin-dependent protein kinase II (CaMKII) [47,48], protein kinase C (PKC) and protein kinase A (PKA). In this sense, AC2, AC3, and AC5 can be stimulated by PKC, while AC6 activity is inhibited. PKA phosphorylation inhibits the activities of AC 5, 6, and 8. However, the main regulatory mechanism is Ca^2+^, which following binding to CaM, may leads to a stimulation of AC1 and AC8, and CaM-independently inhibition of AC5 and AC6; and inhibition of AC3 by CaMKII phosphorylation [40,49].

Regarding the synthesis of cGMP, in vascular SMCs two types of GC synthesize cGMP: a particulate or membrane form (pGC) and a soluble or cytosolic form (sGC) [9,10]. The two ways to convert GTP into cGMP in the presence of Mg^2+^ and are regulated differently, with sGC being activated by NO and by NO donors [50,51,52] and pGC by natriuretic peptides, such as atrial (ANP), brain (BNP), and C-type natriuretic peptide (CNP) [51,53,54]. The activation of each GC is involved in cGMP signaling [9,55,56,57] and can lead to different effects that can be explained based on the compartmentation of cyclic nucleotides [10], which is the main focus of this review.

**Figure 2:**
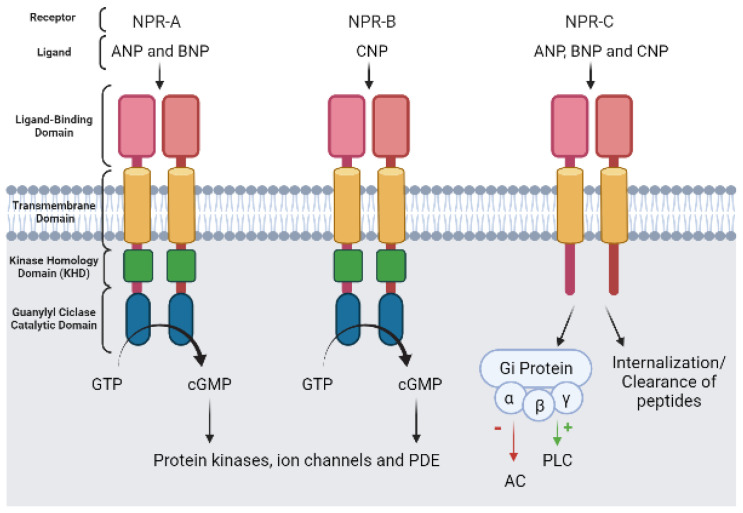
Structure of the natriuretic peptide receptors (NPR) (created with biorender.com). The NPR type A (NPR-A) and NPR type B (NPR-B) receptors have a similar structure, which consists of the extracellular N-terminal domain, the transmembrane domain, the kinase homology domain (KHD), the domain responsible for dimerization, and the GC catalytic domain, which contains the C-terminal region. The NPR type C (NPR-C) receptor has extracellular N-terminal and transmembrane domains but lacks the GC catalytic domain.

The particulate guanylyl cyclase (pGC-A to pGC-G) is a single-chain haem-free transmembrane protein [9,58]. In vascular SMCs, three isoforms of pGC have been identified, in which binding of is NP activated. They are also referred to as natriuretic peptide receptors (NPR): pGC-A (or NPR-A), pGC-B (or NPR-B), and the clearance receptor (or NPR-C) (Figure 2) [9,54]. The NPR-C receptor is the most abundant isoform, and in the cardiovascular system is mainly expressed in the atrium, vascular smooth muscle, and endothelium [59]. However, NPR-A and NPR-B are also highly expressed in vascular SMCs [60,61]. 

The NPR-A and B receptors are transmembrane homodimers composed of an N-terminal extracellular portion, a short α-helical hydrophobic transmembrane chain, a juxtamembrane domain, a kinase homology domain (KHD), a hinge region, and a C-terminal catalytic domain. The extracellular N-terminal region represents the ligand binding domain, showing the highest diversity among the seven pGC isoforms [62]. The NPR type C (NPR-C) receptor has an extracellular domain, lacks guanylyl cyclase activity, and is viewed as a clearance receptor. However, Gi protein signaling has been associated with this receptor within the intracellular C-terminal tail which couples to adenylyl cyclase inhibition (by Gi α subunit) and phospholipase C-β activation (by Gi βγ subunits) [58,63].

Regarding NP affinity, the atrial natriuretic peptide (ANP) and the brain natriuretic peptide (BNP) can bind to NPR-A and NPR-C, and natriuretic peptide C (CNP) can bind to NPR- B and NPR-C, with the same affinity [9,64]. Concerning the main function of the NP in the vascular system, the CNP has been involved in the control of vascular tone, smooth muscle and endothelial cell proliferation, vascular integrity, coronary blood flow and atherosclerosis [65]. The ANP and BNP have demonstrated properties leading to vascular relaxation [58,66]. 

The soluble guanylyl cyclase is a heterodimeric protein complex composed of one α-subunit and one β-subunit. Theoretically, the association of α and β subunits could give rise to at least four different isoforms, but only the α2β1 and α1β1 isoforms (recent nomenclature NO-GC2 and NO-GC1, respectively) are reported to be active [67]. Both α2β1 and α1β1 have similar sensitivity to NO and to drugs that modulate NO-GC activity [66]. In vascular tissue, the α1β1 subunits are predominant [68]. This protein may be linked to a heme prosthetic group through histidine 105 of the β1 subunit [69] which allows NO to bind [70]. The formation of the NO-heme complex and the subsequent conformation change of this complex [71] is responsible for increasing the catalytic activity of this enzyme by approximately 200 times [67,70]. Both subunits of the heterodimer have a similar structure, a regulatory domain linked by the heme group in the N-terminal H-NOX domain, two central domains (a Per/Arnt/Sim (PAS) domain and coiled-coil (CC) domain), and a highly conserved C-terminal catalytic domain [9,70]. Two years ago, in 2019, the molecular structure of the sGC was elucidated by cryo-electron microscopy (cryo-EM) [72,73] allowing us to discover that the central domains are the mediators by protein-protein interactions, while enzymatic activity occurs at the C-terminal catalytic domain. The N-terminal H-NOX domain, since it has a heme group, facilitates the high-affinity binding of NO [73].

The sGC are stimulated by NO and by the carbon monoxide (CO) [58]. NO is synthesized from the oxidation of the amino acid L-arginine, which is catalysed by the nitric oxide synthase (NOS), with simultaneous formation of L-citrulline [55]. Moreover, an additional crosstalk mechanism was observed between NO and hydrogen sulphide pathways that modulate sGC redox [74]. Three NOS isoforms were cloned and characterized; two are Ca-CaM dependent and are constitutively expressed, neuronal nitric oxide synthase (nNOS or NOS1) and endothelial nitric oxide synthase (eNOS or NOS3). The other isoform of NOS is inducible (iNOS or NOS2) and independent of Ca^2+^ [75]. In the active form, all isoforms are homodimers, with each subunit having two domains joined by the binding site of the Ca-CaM complex [76]. Regarding nNOS, this is expressed in the endothelium and SMCs of several types of vessels. Thus, this isoform seems to exhibit an important physiological role in the control of vascular homeostasis [77]. eNOS is the predominant NOS isoform in the vasculature and is responsible for most of the NO produced effect in the vascular system [78]. However, in response to inflammatory cytokines, the vascular smooth muscle expressed the predominant isoform iNOS. In this sense, the expression of iNOS seems to be associated with the development of several pathologies, mainly in atherosclerosis [79]. 

NO is a typical example of paracrine communication since it is synthesized and affects the adjacent cell. Thus, at the vascular level, immediately after its synthesis in the cytosol of endothelial cells (EC), NO crosses cell membranes and binds and activate sGC in vascular SMCs, increasing cGMP synthesis [75,80,81]. On the other hand, some authors showed NO-independent sGC activators/stimulators. These compounds can be classified into two classes: (1) compounds that act as NO-independent but haem-dependent stimulators of sGC, and (2) compounds that acts NO-independent and haem-independent sGC activators. This second class of compounds are termed ‘sGC activators’ to discriminate them from ‘sGC stimulators’ [82,83]. Overall, all these compounds play a critical role at the pharmacological level, allowing unravelling of the physiology and pathophysiology of the NO/sGC/cGMP pathway [83], as explained in more detail under the heading “Compartmentation as a potential therapeutic approach for CVD”.

## 4. Vascular Function of Cyclic Nucleotide Phosphodiesterases

PDEs are a large gene superfamily of isozymes that catalyse the hydrolysis of the 3′ cyclic phosphate bonds of cAMP and cGMP [5,11,19]. Thereby, PDEs produce the inactive metabolites 5′-AMP and 5′-GMP, respectively [19,84], limiting the activity of these molecules on their substrates (protein kinase A or cAMP-dependent protein kinase, PKA and protein kinase G or cGMP-dependent protein kinase, PKG) [5,19]. However, PKA and PKG are not exclusive effectors; cAMP also acts by exchange protein directly activated by cAMP (EPAC) and Popeye domain-containing proteins (POPDC). Both cyclic nucleotides activate cyclic nucleotide-gated (CNG) channels and modulate specific PDEs. In vascular tissues, the concentration of cAMP is usually about five times higher than cGMP, although levels can have significant variability [7].

The varied subcellular localization of PDE is key for the spatiotemporal regulation of cyclic nucleotide-dependent signalling [11]. The relative contribution of each PDE is variable and depends, among other factors, on species, the vascular bed, and the status of the cells [5,19,85]. Therefore, the PDE plays a fundamental role in the generation of specific physiological responses [84], namely in the modulation of signal transduction in blood vessel by a crosstalk between NO and cyclic nucleotide phosphodiesterases [86]. Overall, PDEs display a common general structure with an isoform-specific N-terminal regulatory domain, a conserved catalytic domain (25–52% homology), and a C-terminal domain. The regulatory domains contain various structural features involved in the regulation, binding of regulatory molecules, localization, and dimerization of the enzymes [87].

Eleven PDE families have been identified [88,89] that are encoded by 21 genes and can give rise to around 100 isoforms [19,90]. The multiple PDE isoforms have different functions and structures [89], but several studies agree that their main function is the regulation of cyclic nucleotide (cAMP and cGMP) pools controlling multiple cellular processes [5,19,89]. The PDE isoforms are classified according to a common nomenclature: each PDE isoform has a family number (1-11), each PDE is represented by a capital letter indicating the gene (A-D), and a final number corresponding to the splice variant [19,91]. The PDE isoforms can also be classified on the basis of the specificity of their hydrolysis [19,89]: (1) PDEs highly specific for cAMP hydrolysis are PDE4, PDE7 and PDE8; (2) PDEs highly specific for cGMP hydrolysis are PDE5, PDE6 and PDE9; (3) PDEs with dual specificity for cAMP and cGMP are PDE1, PDE3, PDE2, PDE10 and PDE11. PDE3 and PDE10 are cGMP-sensitive but cAMP-selective [11,19,66,92]. In vascular SMCs, the main PDEs that hydrolyse cAMP are PDE3 and PDE4 isoforms, while PDE1, PDE3, and PDE5 hydrolyse cGMP [85,93] (Table 1)**.**

The PDE1 family has the only PDEs activated by the binding of the Calcium-Calmodulin (Ca^2+^-CaM) complex. PDE1 is transcribed by three genes (PDE1A, PDE1B, and PDE1C), which give rise to several isoforms [88,91]. The two connection sites for the Ca^2+^-CaM complex are in the N-terminal domain. PKA and PDE1B can phosphorylate PDE1A by CaM-dependent kinase-II; both phosphorylations decrease the activation capacity caused by CaM. Activation of PDE1C can be reduced by PKA [19,84]. Moreover, in arterial SMCs, higher intracellular Ca^2+^ concentrations inhibit AC3 and activate PDE1C [49]. PDE1 is present in a wide variety of vascular SMCs [88,94], including human arteries [6,93,95].

At the vascular level, PDE1C is highly expressed in primary vascular SMCs cultures. In contrast, this PDE is not expressed in human vascular SMCs of the contractile phenotype, allowing the use of this PDE as a marker of human SMC proliferation. In the human vasculature, PDE1C appears to be exclusively functional in developing these cells during normal vascular development and pathological vascular remodeling [96]. The fact that this PDE is only present in synthetic SMCs under pathological conditions (and not in normal vasculature), suggests that PDE1C inhibitors may be focused on disease processes and do not interfere with normal vascular function. Chan et al. (2011) showed that an increase in PDE1C activity contributes to decreased cAMP and increased proliferation of pulmonary artery SMCs in patients with pulmonary hypertension [96]. Concerning the PDE1A and PDE1B, both are expressed in human SMCs with the contractile phenotype, while in the synthetic phenotype only PDE1A is present [97]. Full activation of PDE1 occurs only after Ca^2+^ and calmodulin binding. Some vasoconstrictors, namely norepinephrine, angiotensin II (Ang II), and endothelin-1, increase intracellular Ca^2+^ levels inducing PDE1 activation. In this process, due to a decrease in the cGMP levels, the vasoconstrictor effect induced by vasoactive agents is increased [66]. Thus, PDE1 isoforms may be good therapeutic targets for both primary and secondary forms of the disease. Selective PDE1 inhibitors appear to be particularly attractive as novel therapeutics to attenuate the pathophysiological abnormalities that occur in pulmonary hypertension [98]. Moreover, recent data suggest that inosine 3′,5′-cyclic monophosphate (cIMP) levels in coronary arteries are regulated by PDE1 and PDE5, whose inhibition at a certain level led to increased cIMP content that may improve hypoxic constriction [99].

The PDE2 family (initially named cGS-PDE) exhibits similar hydrolysing maximal velocities and Km for both cGMP (10 µM) and cAMP (30 µM) [19]. In mammalian species, PDE2 is only transcribed by a single gene (PDE2A), and there are three known splice variants: PDE2A1, PDE2A2, and PDE2A3; however, PDE2A2 has only been found in rats. PDE2 is stimulated allosterically by binding cGMP to the regulatory domain. PDE2A1 is present in the cytosol, while PDE2A2 and PDE2A3 are located in the plasma membrane. The different localization of these variants is probably due to a unique N-terminal sequence, which is absent in PDE2A1 [92]. PDE2 was first shown to be expressed in EC [100] and to participate in endothelial proliferation [101]. Moreover, PDE2 expression was found in pulmonary artery SMCs but only from patients with pulmonary hypertension (PH) [98], pulmonary arterial hypertension (PAH) [102] and in pulmonary arteries from rats with hypoxia-induced PH [102]. However, the presence of this PDE in SMCs under physiological conditions continues to be doubtful. 

The PDE3 family displays dual specificity with high affinity for both cAMP and cGMP but with much higher turnover rates for cAMP [9,19]. Furthermore, PDE3 shows a relatively high affinity for cGMP, which acts as a competitive inhibitor of cAMP hydrolysis, creating the so-called positive cGMP-to-cAMP crosstalk. PDE3 isoforms are transcribed by two genes (PDE3A and PDE3B). To date, there are three isoforms described (PDE3A1-3) and only one PDE3B isoform. However, in the cardiovascular (CV) system, mainly in the vascular SM, PDE3A is the predominant isoform found, while PDE3B is more highly expressed in cells involved in the regulation of glucose and lipid metabolism [103]. Regarding the expression of PDE3 in the two vascular SMCs phenotypes, both contractile and synthetic express both PDE3 gene products (PDE3A and PDE3B), but PDE3A is substantially lower in synthetic than in contractile vascular SMCs [104]. PDE3 can be activated by PKA and protein kinase B [90,104] and is very important in the contractile phenotype, but its activity is decreased in the synthetic phenotype [105]. Moreover, PDE3 isoforms are important in several CV physiological processes such as blood pressure regulation and vascular smooth muscle reactivity [106]. Recently, Ercu et al. 2020 showed that a mutated PDE3A gene drives mechanisms increasing the peripheral vascular resistance which may lead to hypertension. The authors suggest a gain of function mutation related to hypertension associated with brachydactyly [107]. 

The PDE4 family is highly specific for cAMP hydrolysis and is inhibited by rolipram, a drug that is used to distinguish it from other PDEs [19]. These cAMP-specific PDE4s are the largest family with over 20 isoforms encoded by four genes (A, B, C, and D). Overall, each isoform has an N-terminal targeting domain, containing upstream conserved regions 1 and 2 (UCR1 and UCR2). These regions, when present, are coupled to the catalytic domain by linker regions 1 and 2 (LR1 and LR2). Thus, based on their presence and the size of UCR1 and UCR2, the various PDE4 isoforms can be categorised into (1) long isoforms with UCR1 and two present; (2) short isoforms with only UCR2 present; (3) super-short with a truncated UCR2, and (4) dead-short isoforms without both UCR domains, but with a truncated catalytic domain [108,109,110]. The capacity of PDE4 isoforms to be regulated by phosphorylation through the action of a variety of protein kinases is determined by the various UCR1/2 combinations; this interaction allows the PDE to have a major role in the integration of the responses between different signaling pathways [111,112]. At the level of vascular smooth muscle, its expression was observed in the aortas of bovine [93], pig [113] and rat [93], in rat mesenteric [114], human pulmonary [115] and human umbilical artery [6,101]. At the vascular level, inhibition of this PDE4 generally causes vasorelaxation, and some authors consider it to be the most important PDE in the regulation of vasodilation associated with cAMP, namely, in the canine basilar artery [116] and the human umbilical artery [6,101]. Regarding vascular SMCs, the expression of PDE4A and PDE4B, as well as numerous variants of PDE4D, has been observed [117]. Moreover, PDE4D is the predominant cAMP-degrading isoform in cultured vascular SMCs; the prolonged cAMP elevation increases PDE4D activity, and the phenotype stage of the cell is modulated by PDE4D variants [118].

The PDE5 family is characterized by cGMP-specific hydrolysis encoded by just one gene (PDE5A), with three known isoforms (PDE5A1-3). Although these three isoforms differ from one another in the C-terminal domain, to date, no differences in their functionality have been detected [119,120,121]. The PDE5 is a multidomain protein [84] and structurally is constituted by a catalytic domain, which is located at the C-terminus of the protein, and a regulatory GAF domain (GAFa-GAFb) in tandem in the N-terminal side part of the molecule. The N-terminal GAFa domain binds cGMP and allosterically modulates the catalytic activity. In contrast, the C-terminal GAFb domain plays a role in dimerization of the enzyme [122,123]. On the other hand, the GAF domains near the N-terminal regulatory region, have a PKG phosphorylation site which is conserved across all PDE5 isoforms [84,94]. The PDE5 is the most abundant and active cGMP-PDE in the vascular SMCs [85,94], and the two vascular SMCs phenotypes present the same PDE5 expression [124]. PDE5 is the key PDE in the regulation of vasodilation associated with cGMP in arteries [6,11,89,115,125] and in regulating cGMP pools in both contractile and synthetic phenotypes [126].

The PDE7 family is specific for cAMP hydrolysis and is encoded by two genes (PDE7A and PDE7B), which allow the expression of several isoforms [127]. PDE7A present three isoforms (PDE7A1, PDE7A2, and PDE7A3) but, in contrast, PDE7B exists as a single isoform in humans [127]. The PDE7 expression was reported in several cultured vascular SMCs [128,129], the expression of PDE7A being dominant in adult rat aortic smooth muscle cells [129]. In addition, a recent study observed an increase of PDE7B expression in the high-density culture (contractile phenotype) of rat aortic SMCs (RASMCs) [130]. However, its activity was uncertain [129]. In humans, PDE7A activity in the pulmonary artery [19,94] was demonstrated. Smith et al. (2003) also reported that the isoform PDE7A1 (but not PDE7A2) is present in human vascular SMCs [127].

The PDE8 family is one of the most recently discovered families. These PDEs are highly specific for cAMP hydrolysis and do not hydrolyse cGMP, nor are they regulated by cGMP [131]. PDE8 is transcribed by two genes (PDE8A and PDE8B). Recently the, PDE8A mRNA expression in RASMCs has been observed, but PDE8B was not present. However, mRNA expression encoding PDE8A was significantly decreased in a high-density culture of RASMCs compared to low-density culture [130].

The PDE9 family is highly specific for cGMP hydrolysis and shows a higher affinity for cGMP (Km = 0.07–0.17 μM) than all members of the PDEs superfamily [132]. PDE9A is the only gene; however, more than 20 variants have been observed suggesting that this gene may have complex expression regulation [19,133]. Until recently, nothing was known about the role of PDE9 in the CV system, although it is expressed at the mRNA level in confluent cultured rat pulmonary and systemic coronary SMCs [128]. Recent data have shown that this PDE may be involved in vascular compartmentation [126].

Concerning the PDE10 family, these are PDEs with dual specificity once they hydrolyse both cAMP and cGMP. A single gene, PDE10A, encodes this PDE10, and two major variants, PDE10A1 and PDE10A2, and several minor PDE10A variants have been described in humans [90]. The PDE10A GAF-B domain is the only one modulated by cAMP [134]. In addition, some authors demonstrated the PDE10A expression in SMCs of the pulmonary artery (layers and cells in contractile phenotype). Moreover, the same authors suggested that this PDE plays a central role in progressive pulmonary vascular remodelling and suggested the use of an inhibitor of PDE10A as a novel therapeutic approach to PAH treatment [135].

## 5. How Do Cyclic Nucleotides Regulate Vascular Tone?

**Figure 3:**
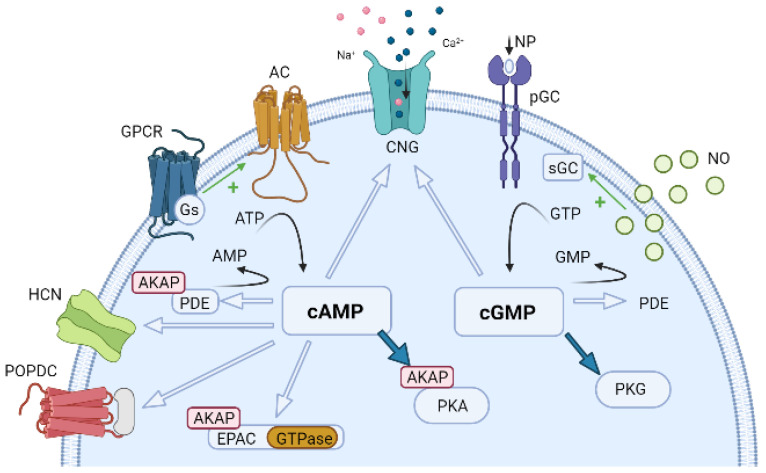
Cyclic nucleotide effectors in the vascular smooth muscle (created with biorender.com). cAMP is synthetized by transmembrane adenylyl cyclases (AC, activated by Gs proteins) and acts mainly by protein kinase A (PKA) and by an exchange protein directly activated by cAMP (EPAC) and Popeye domain-containing proteins (POPDC). cGMP is synthetized by particulate GC (activated by natriuretic peptides, NP) and by soluble GC (activated by nitric oxide, NO) and acts mainly by protein kinase G (PKG). Finally, both cyclic nucleotides activate cyclic nucleotide-gated (CNG) channels and modulate specific cyclic nucleotide phosphodiesterases (PDEs).

Several mediators and drugs regulate blood flow and blood pressure. The cyclic nucleotides cAMP and cGMP are the key messengers mediating vasodilation under physiological conditions and are therefore involved in the physiological regulation of vascular tone. Their function is due to the existence of several mechanisms that include the reduction of [Ca^2+^]_i_ and a decrease in the sensitivity of the contractile machinery; both mechanisms may occur together and decrease MLC phosphorylation. On the other hand, these mechanisms are regulated by several cellular effectors, including ion channels [7] that regulate the membrane potential and Ca^2+^ influx. All these mechanisms promote vessel relaxation by reducing vascular contractility and tone [5,8]. In this review, the critical cAMP and cGMP effector proteins are addressed, and Figure 3 represents the main pathway involved by these effectors in vascular tone.

### 5.1. cAMP Effector Proteins

cAMP is generated in response to the activation of a wide range of membrane receptors belonging to the GPCR superfamily. After binding a ligand, a G-protein is activated and promotes the activation of the AC that generates cAMP from ATP. This second intracellular messenger, in turn, regulates an infinity of physiological and pathological processes in different organs, including the CV system, and this is achieved by activating the so-called cAMP effectors. These effectors include cAMP-dependent protein kinase (protein kinase A, PKA), exchange protein directly activated by cAMP (EPAC), cyclic nucleotide-gated (CNG) ion channels, and the recently discovered Popeye domain-containing proteins (POPDC) [136].

The major effects of cAMP in eukaryotic cells result from the activation of PKA [5]. PKA was first discovered by Walsh et al. in 1968 [137]. Its catalytic subunit (C) was the first protein kinase to be crystallized. Since then, many studies have focused on it, and is currently one of the most studied protein kinases. However, and despite intense research, some important aspects such as its activation and inactivation in the complex cellular environment still need to be investigated [138].

Structurally, the PKA holoenzyme is a tetramer formed by two catalytic C subunits, and a dimer regulatory (R) subunit, where cAMP binds [139]. The three isoforms of the C subunit (α, β, γ) have virtually identical kinetic and physicochemical properties, while the four regulatory subunits (RIα, RIIα, RIβ, and RIIβ) exhibit distinct binding affinities for cAMP and are differentially located within cells [139,140,141,142,143]. PKA holoenzymes containing the RI subunits are PKA type I (RIα and RIβ) and are predominantly in the cytoplasm. In contrast, PKA holoenzymes containing type II subunits are called PKA type II (RIIα and RIIβ) and appear to be associated with cellular structures and organelles [139,143]. Initially it was thought that this could be due to the anchoring of A-kinase anchoring proteins (AKAP) with greater affinity for the RII subunits [139]. Still, other studies have demonstrated that there are AKAP with dual specificity for the RI and RII subunits and AKAP that bind only to the RI subunits. The studies show both PKA type I and II may be anchored in subcellular compartments within the cells [143,144,145,146] and, therefore, the specificity of PKA signaling is achieved by binding to these anchoring proteins [147,148,149]. Overall, AKAP direct holoenzymes PKA to different subcellular sites near neighbouring proteins, optimizing signal transduction and allowing events responsive to local cAMP to occur within specific compartments of the cell (i.e., resulting in compartmentalized cAMP signaling, explained in more detail below) [149,150]. Thus, an imbalance in the expression or activity of some of the PKA subunits [151,152] or anchoring by AKAP [153] may lead to the development of cardiovascular diseases (e.g., myocardial infarction). 

In the absence of cAMP, PKA is in an inactive state, with two regulatory subunits, R, and two catalytic subunits, C, forming a complex called a tetrameric holoenzyme (R2C2) [154]. In each regulatory subunit R, there are two binding sites (A and B) to which cAMP cooperatively binds during activation [143]. The inactive PKA holoenzyme exposes the available B site for cAMP binding. When occupied, the binding of cAMP to site A is increased, which leads to an intramolecular conformational change: the regulatory subunits dissociate from the R2C2 complex and generate two active C subunits, which phosphorylate their substrates in the cytosol and the nucleus [143,149,155,156,157,158]. Importantly, only the C subunit is in the nucleus [159]. In other words, the binding of the C subunit to the inhibitory sites of the respective R subunit renders the kinase-inactive, while the cAMP allosteric binding to two C-terminal tandem cAMP binding domains (CNB-A and CNB-B) of the R subunits triggers the catalytic activity of the holoenzyme [160,161]. However, it has been suggested that cAMP can also activate PKA without releasing the catalytic subunits C [138,162,163,164] and there are intact and active holoenzymes within the cytoplasm in the presence of cAMP. Other studies suggest that a more significant number of R subunits than C are essential for reducing the release of catalytic C subunits and increasing their recapture [159]. 

PKA may also play a critical role in the phenotypic modulation of the vascular SMCs, that mainly depend on intracellular ATP concentrations. Hogarth at al demonstrated that low micromolar ATP concentrations induce a negligible effect on DNA synthesis but induce higher serum response factor activity, and showed a higher gene expression of the markers of differentiated smooth muscle cells, such as SM-α-actin and SM22, indicating a contractile SMCs phenotype. On the contrary, high micromolar ATP concentration inhibits serum response factor activity and the markers genes expression and promotes cultured cell growth in a manner dependent on PKA activation. The change into the contractile phenotype by high ATP intracellular can be prevented, and even reversed, by inhibition of PKA activity [165]. 

Although PKA is the primary protein kinase activated by cAMP, several studies suggest that some of the biological effects of cAMP may, at least in part, be mediated by activation of PKG [166,167,168] which is classically activated by cGMP. The cyclic nucleotide-binding domain of PKG binds cGMP with greater affinity than cAMP. Moreover, intracellular concentrations of cAMP are generally higher than cGMP, which show that cAMP is also an agonist of PKG [169]. In this sense, Eckly-Michel et al. (1997) showed clearly that cAMP mediates PKG activation in vascular smooth muscle [170]. 

In addition to PKA, another important downstream effector of cAMP [171] is the EPAC family [137,172,173]. The EPAC proteins (1 and 2) act as guanine nucleotide exchange factors (GEFs) for small Ras-like GTPases (Rap1 and Rap2), and for this reason are also designated as cAMP-GEF proteins. The EPAC1 encoded by the RAPGEF3 gene is present in the human vasculature [171]. Although EPAC and PKA can act independently, they often act together in the same biological process in which they mediate synergistic or opposite effects. Discovery of EPAC discovery is relatively recent, but it modulates several different systems, including the CV system, which plays a critical role [171]. Normally, in vascular SMCs, activation of RhoA/Rho kinase (ROCK) signalling phosphorylates MLCP, inhibiting its phosphatase activity. Thus, phosphorylation of MLC by MLCK increases, activated by the Ca^2+^-CaM complex, and contraction occurs. EPAC induces a vasorelaxation of vascular SMCs through inhibition of RhoA/ROCK signalling. The cAMP-mediated activation of EPAC/Rap1 releases the inhibitory effect of RhoA/ROCK on the MLCP. In turn, this leads to dephosphorylation of MLC and subsequent vasorelaxation of SMCs [171]. However, the role of EPAC in the vasculature is controversial: these proteins may promote or inhibit SMCs proliferation and migration. Moreover, if EPACs induce vasorelaxation of vascular SMCs extracted from large vessels, on the other hand they induce vasoconstriction of micro vascular SMCs. In other words, EPAC proteins may act as mediator of vascular SMCs phenotypic switching or, on the other hand, have a protective role [171]. This disagreement of the effects of EPACs seems to be based on the use of different cellular model systems, as it is known that the susceptibility of vascular SMCs to phenotypic switching is variable according to their origin, the vascular bed from which they were isolated, as well as differences in species, strain, age, or sex of the animal model. In this sense, there is no doubt that their role in the vasculature is crucial, and that anomalous EPAC signaling may be frequently implicated in pathological CV conditions [174]. Therefore, Rap1-independent EPAC signalling may be a useful target for drugs designed to treat atherosclerosis and hypertension.

Additionally, other effectors of cAMP are CNGs, expressed in vascular SMCs [175,176] and EC [176,177,178]. CNGs are nonselective cationic channels that open in response to direct binding of cAMP and cGMP [179], modulating the vascular tone. 

Moreover, POPDC also play the role of cAMP effectors. The POPDC family of proteins (1-3) contain an extracellular N-terminal domain, three transmembrane domains, and a cytosolic Popeye domain [180]. This latter domain serves as the binding site for high-affinity cAMP. POPDC is expressed in vascular SMCs colocalized with α-smooth muscle actin and appears to be involved in the mechanisms of vasculogenesis [180,181]. However, its role as a cAMP effector in vascular SMCs remains unclear. 

### 5.2. cGMP Effector Proteins

cGMP concentration is critical for the maintenance of CV homeostasis in several cell types. Moreover, the classical and significant physiological effect of an increase in the intracellular concentration of cGMP in vascular cells is vasodilation [5]. The cGMP pathway begins with the activation by one ligand (NO, ANP, BNP, and CNP) to guanylyl cyclase, either soluble or particulate, that induces the increase of the intracellular concentration of cGMP, which may activate three classes of cGMP effector proteins: (1) PKG, (2) CNG channels and (3) PDE, whose activity can be regulated by cGMP as previously described [9,19,123].

This protein kinase (also known as cGKs) alters the activity of target proteins, phosphorylating specific radicals of serine and threonine [182,183]. Moreover, PKG is considered the most important target of cGMP in the CV system. In mammals, two PKG genes have been identified, namely *PRKG1 AND PRKG2*, that encode PKG1 and PKG2, respectively [184,185,186]. The membrane PKG 2 is not expressed in the CV system. On the other hand, cytosolic PKG 1 has two different splice variants (PKG 1α and PKG 1β) that differ only in the first~100 amino acids. However, the one most expressed in vascular SMCs is PKG 1β [184,187,188,189]. In these cells, both PKG 1α and PKG 1β are expressed, while EC expresses only PKG 1β [9]. However, the expression of PKG is dependent of the cell density in culture, low density, or reduced expression of PKG [190]. Moreover, the increase in the expression of PKG contributes to change for the contractile phenotypic in cultured vascular SMCs, and the suppression of PKG expression during cultured growth in vitro may facilitate the modulation to a more synthetic, dedifferentiated phenotype [191]. The crystal structure of PKG 1 was elucidated only in 2016 [192]. PKG1 consists of a homodimer divided into a regulatory (R) and a catalytic (C) region. The regulatory region is composed of four functional domains, leucine zipper, auto-inhibitory (AI) (which engages the catalytic region of the PKG in the committed/inactive state), and two tandemly arranged low and high-affinity cGMP binding domains (CNB-A and CNB-B) [192,193]. Selective interaction of cGMP with the cyclic nucleotide-binding domain binding pockets leads to a 10-fold higher sensitivity for PKG 1α compared to PKG 1β [194]. Two domains constitute the C-terminal catalytic region, the kinase domain (where the Mg^2+^/ATP binds) and the AGC-kinase C-terminal domain (where substrate/downstream proteins bind and phosphorylation occurs) [193]. This protein can bind or activate several signaling pathways, but the exact mechanisms underlying their interactions with PKG have not yet been elucidated. Briefly, PKG operates in parallel through several mechanisms to reduce vascular contractility: (1) PKG inhibits IP3 stimulated SR Ca^2+^ release by the IP3R by phosphorylating the IP3 receptor-associated PKG-substrate (IRAG) [195,196]; (2) PKG can inhibit by direct phosphorylation the IP3R [196]; (3) PKG phosphorylates phospholamban which removes the brake on SERCA activity, increasing Ca^2+^ import into the SR; (4) IP3 concentration due to phospholipase C activation is suppressed by PKG phosphorylation of the regulator of G-protein signalling 2 (RGS2) [197]; (5) PKG prevents the inactivation of MLCP by ROCK [198,199]; (6) PKG phosphorylate Ser-695 on the myosin phosphatase target subunit 1 (MYPT1) of the MLCP, which prevents ROCK from phosphorylating the adjacent Thr-696 that usually leads to enzyme inactivation [200,201]; (7) PKG phosphorylates the small heat shock protein HSP20 which induces vascular relaxation (by arresting the reorganization of the actin cytoskeleton required for vasoconstriction) and inhibits of agonist-induced constriction [202,203]; (8) PKG activates large conductance Ca^2+^-activated K^+^ channel (BK_Ca_) [204]; (9) PKG activate Kv [204], and (10) PKG inhibits the voltage-dependent Ca^2+^ channels (see review [7]).

Concerning the CNG channels, these channels are nonselective cation channels activated by the binding of cGMP or cAMP [179], as previously mentioned for cAMP effector proteins. The CNG channels reveal a higher sensitivity for cGMP than for cAMP. In physiological conditions, CNG channels carry inward Na^+^ and Ca^2+^ currents [205]. Even if the divalent cations can permeate the channel, higher concentrations induce these cations to bind to specific sites within the channel pore and block further ion flow. Knowledge of CNG channels is greater regarding the visual and olfactory systems while in other areas is less. Despite that, the CNG channels are used with the patch-clamp recordings to observe cyclic nucleotide changes [206].

Finally, the activity of PDE can be regulated by cGMP [9] as described in Section 4. In addition to PDE, there are crosstalk mechanisms between both cAMP/cGMP pathways [19] which are described detailed in the following section.

## 6. Compartmentation of Cyclic Nucleotides Signaling

The understanding of vascular cyclic nucleotide signalling is fundamental, since the actions of the local cyclic nucleotide and the interaction of the two signalling pathways are implicated in (patho)physiological conditions.

Compartmentalized cyclic nucleotide signaling involves multiple receptor stimuli triggering diverse intracellular effects generated by producing just a few second messengers of cAMP and cGMP. The term “compartmentation” refers to the mechanisms by which multiple spatially segregated cAMP/PKA and cGMP/PKG signalling pathways exert different, or even opposite, functional effects on distinct subcellular microdomains of the same cell [5,18,19]. 

Many years ago, PDE-mediated hydrolysis of cyclic nucleotides was widely studied as one of the mechanisms involved in the compartmentation of cyclic nucleotides [6,10,11,111]. PDE control the compartmentation of the cyclic nucleotides by their local hydrolytic degradation and also by creating different concentrations of cAMP and/or cGMP in different cytosolic sites [5,207]. Therefore, PDE are currently of high pharmacological and clinical interest (for a better understanding please see the reviews [11]). 

In addition to PDE, a number of other proteins appear to be involved in the compartmentation of nucleotide cycles, such as GPCR [208], AC and GC [209], scaffold proteins [210,211] including AKAP [11,212] and Caveolin-3 [213] and also, physical barriers such as the nucleus, sarcoplasmic reticulum and mitochondria (see the review [214]). Moreover, there is multidrug resistance-associated protein 4 (MRP4), a member of a large family of transmembrane proteins involved in active transport of substrates out of cells, which pumps cyclic nucleotides and controls multiple cardiovascular processes (e.g., endothelial barrier function, vascular SMCs proliferation and vasodilation) [215]. Sassi et al. (2008) were the first authors to identify MRP4 in vascular smooth muscle as a regulator of SMCs proliferation [216]. The authors observed that these energy-dependent efflux pump are modulators of signal transduction mediated by cyclic nucleotides (cAMP and cGMP) in human coronary artery SMCs. The inhibition of MRP4 modified the intracellular content of cyclic nucleotides and markedly enhanced their antiproliferative effect. It is unclear how the MRP4 inhibition modulates the PDE expression and/or activity, except that PDE activity does not counteract the effects induced by MRP4 inhibition. Moreover, MRP4 inhibition alone seems to be sufficient to modulate intracellular cyclic nucleotide levels and signal transduction [216]. In this sense, MRP4 may be an alternative or complement to PDE, ensuring intracellular cyclic nucleotide homeostasis. MRP4 inhibition may have therapeutic implications in vasculoproliferative disorders and protect from pulmonary hypertension [215]. However, the role of MRP4 for the compartmentation of cyclic nucleotides in vascular SMCs remains unclear. 

Additionally, there is a crosstalk between cyclic nucleotide signaling pathways that is very complex and regulated differently by various factors in different types of cells in the CV system, and by distinct subcellular microdomains (as detailed described in review [19]). Briefly, also in this process, the presence and activity of PDE in a specific microdomain influence the local levels of cyclic nucleotides, playing a fundamental role in their compartmentalized signaling [217]. 

**Figure 4:**
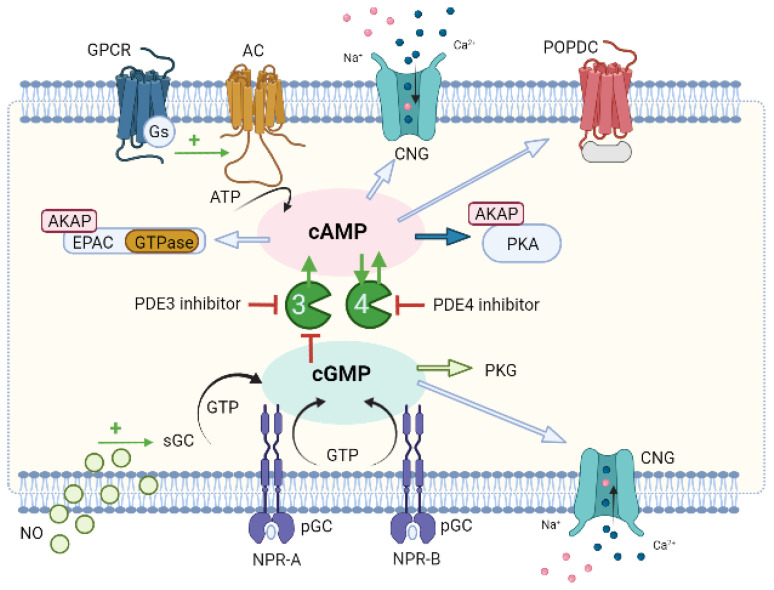
Schematic representation of crosstalk cAMP/cGMP signaling mediated by cyclic nucleotide phosphodiesterases (PDEs) (created with biorender.com). In vascular SMCs, NO induces crosstalk mechanism between PDE3 and PDE4 that mediates vasodilatation.

In vascular SMCs, NO induces a crosstalk mechanism between PDE3 and PDE4 that mediates vasodilatation (Figure 4). Briefly, PDE3 because it has a higher Vmax/Km ratio than PDE4 controls basal cAMP levels. Thus, in the absence of endothelium, an increase in cGMP by an NO donor (which stimulates GC), or a PDE3 inhibitor (cGMP inhibits PDE3) causes an increase in cAMP concentrations, triggering PDE4 to regulate its signalling. When PDE4 is inhibited, cAMP levels increase and vasorelaxation of SMCs occurs. On the other hand, when the endothelium is functional, NO is produced by sGC, and an increase in cGMP levels occurs. This increase also inhibits PDE3, and so it is, again, PDE4 that controls cAMP regulation [19]. 

Moreover, the β-adrenergic and NO/cGMP/PKG pathways are important to control the molecular selectivity of PDE to cyclic nucleotides. Recently, Dillard et al. (2020) reported in human pulmonary artery SMCs that the cGMP pathway increases AMPK phosphorylation by a PDE3A-mediated mechanism [218].

Over the years, several techniques have been developed to study the compartmentation of cAMP and cGMP. Among them are radio and enzyme-linked immunoassays (biochemical techniques) and electrophysiological approaches such as the patch-clamp technique. These techniques, despite being very sensitive and specific, are limited in their capacity to record and analyse cyclic nucleotide gradients directly in subcellular microdomains under physiological conditions [219]. Innovative techniques, such as fluorescent energy transfer resonance (FRET), cell transfection with CNG channels, the development of genetically encoded fluorescent biosensors for cGMP and even the combination of FRET and SICM (scanning ion conductance microscopy) techniques, have provided direct evidence of the compartmentation of cyclic nucleotides [10,219,220,221]. Although the study of compartmentation in vascular SMCs is very recent, some studies on this phenomenon at the vascular level are described below.

### 6.1. Compartmentation of cAMP Signalling

cAMP compartmentation is the formation of different subcellular compartments of cAMP signalosomes. Overall, these are multiprotein complexes of key regulators in cAMP signaling, namely: (1) a cAMP effector, mainly PKA, (2) PDE, enzymes responsible for the degradation of cAMP, (3) scaffold proteins, namely AKAP responsible for anchoring the whole complex to a specific subcellular location, and (4) GPCR linked to stimulatory G (Gs) proteins, mainly β-AR (β-adrenergic receptors) located in various membrane structures, which interact with distinct scaffolds and subsets of PDE [159].

Briefly, compartmentalized signaling results from the ability that GPCR gives rise to spatially distinct pools of cAMP. These pools, in turn, activate defined subsets of localized PKA (which are linked to anchoring proteins, AKAP) [222]. The AKAP-mediated signaling organizes within the same macromolecular complex several molecules (GPCR, AC, PDE, PKA, and its targets, and phosphatases) thus guaranteeing selective phosphorylation and very strict local regulation of the duration of the cAMP signal [159,222,223,224,225,226].

According to the literature, more than 50 genes encoding distinct AKAP have been identified. Among the most relevant for effects on the arterial SMC system is an AKAP that anchors PKA to L-type voltage-operated Ca^2+^ channels (L-Type VOCC) at the membrane, having a role in the regulation of vascular function. Recently Nystoriak et al. demonstrated that AKAP5 (referred to as AKAP150 in rodents and AKAP79 in humans) anchored to PKA phosphorylates α1C in Ser1928 of L-Type VOCC, inducing vasoconstriction in diabetes and in response to increased levels of extracellular glucose [227]. Moreover, AKAP5 is vital for the regulation of myogenic tone and for Ca^2+^ sparklets, blood pressure, and the development of hypertension induced by Ang II [228].

On the other hand, PDEs also have an essential role in compartmentalized cAMP signalling, since they are the only way in which cAMP can be degraded in the cell [229,230,231]. PDE establishes boundaries to cAMP diffusion and generates cAMP pools which are confined within subcellular compartments. Therefore, specific subcellular PDE localization is fundamental for the local regulation of the magnitude, duration, and specificity of cAMP signalling. Within the various PDE families, PDE4 is the one that stands out the most in this role [109,232]. A study performed by Richter et al. (2008) also demonstrated that different isoforms of this PDE4 are associated with specific locations of β-AR (1 and 2), supporting not only the compartmentation of cAMP but the existence of other functional effects induced by isoforms of β-AR [233]. 

Regarding the β-AR/cAMP/PDE signaling pathway, it was also recently demonstrated that cell confluency has a critical modulatory role in a study performed with RASMCs [130]. The results obtained by Belacel-Ouari et al. suggest that low cell density is associated with loss of β1-AR membrane expression, less cAMP/PDE activity, and a baseline increase in intracellular cAMP concentration. The authors point out that this can have a pathophysiological impact in disorders involving vascular remodelling [130], highlighted the importance of β-AR/cAMP/PDE signaling. Nevertheless, Dunkerley et al. (2002) observed no differences in PDE4 expression between the two SMCs phenotypes, contractile and synthetic [104]. However, decreases in PDE3A activity and increased expression of PDE1C were related to a synthetic SMCs phenotype [234]. More recently, Zhang et al. (2019) also demonstrated in RASMCs that PDE1 has a small role in controlling cAMP pools. However, the authors observed that the role of this PDE is intensified under high [Ca^2+^]_i_, since they obtained an increase in the activity of this PDE in cAMP signals induced by the stimulation of β-AR in the presence of Ang II [126]. As demonstrated by Kim et al. (2001), this vasoconstrictive agent, Ang II, increases [Ca^2+^]_i_ in cultured RASMCs, allowing stimulation of PDE1 [235]. 

**Figure 5:**
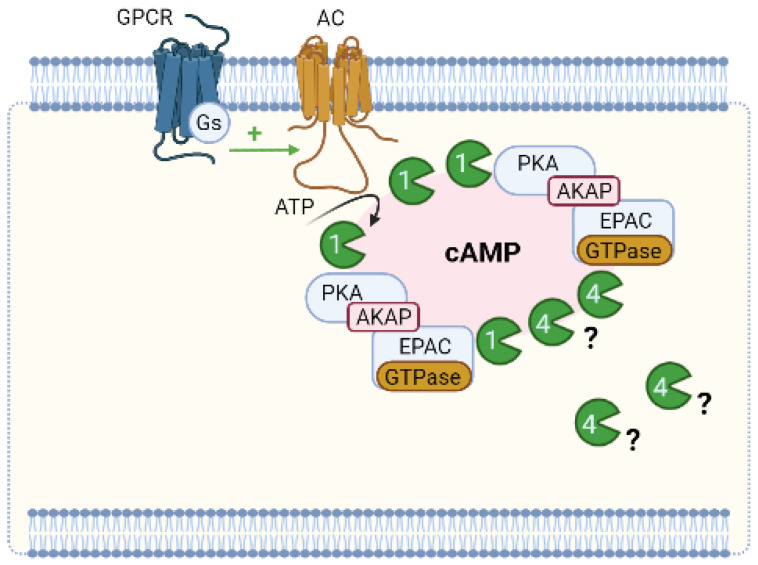
Schematic representation of compartmentation of cAMP signalling in vascular SMCs in a synthetic phenotype (created with biorender.com). In the synthetic phenotype, activating GPCR leads to a pool of cAMP mainly controlled by PDE1.

Although compartmentalized cAMP signaling is currently accepted, and the local subcellular organization and regulation of individual signalosomes are known, aspects of this compartmentation remain to be clarified (Figure 5). One of these aspects is the activation of PKA. Several studies appear to be concessional that, under baseline conditions, localized PDE keeps cAMP concentrations below their activation limits (causing PKA to remain inactive). When there is hormonal stimulation, the production of cAMP increases so much that it exceeds the rate of degradation by PDE, which is present in adjacent to PKA in the nanodomain, thus leading to a transient activation of PKA. PKA phosphorylation of PDE stimulates the hydrolytic activity of cAMP, which leads to a return to baseline levels of cAMP [236]. Thus, PDE, when degrading cAMP locally, determines whether the PKA present in a specific multiprotein complex is activated or not and, therefore, dictates at any time which signalosome is involved in signaling [11]. However, it is questionable how PDEs can keep the cAMP concentration below the PKA activation threshold considering their K_m_ and V_max_ and the PKA activation constant determined in vitro at baseline levels. In addition, these kinetic parameters also raise doubts about how PDEs contribute to the compartmentation of the cAMP response to hormonal stimulation when cAMP levels increase significantly [237]. In this sense, Koschinski et al. (2017) demonstrated that the threshold for PKA activation in intact cells requires higher cAMP concentrations (at least one order of magnitude) than in vitro. The authors explained how PDEs may be able to maintain cAMP concentration below the threshold for PKA activation even at basal cAMP levels and contribute to the compartmentation of the cAMP response to hormonal stimulation when cAMP levels significantly increase [236]. 

Finally, the different subcellular localization of PKA holoenzymes, particularly PKA regulatory subunits, seems to have a fundamental role in the cAMP compartmentation. When the two catalytic subunits are released from the PKA holoenzyme, PKA-RI can become mainly cytosolic, and the PKA-RII can be associated with particulate fraction [159]. Therefore, it seems that depending on the ligand to which the PKA holoenzymes are bound, it may that the catalytic subunits are unable to diffuse within the cell but become constrained by spatial boundaries that lead to the activation of local PKA substrates. However, it has recently been reported that catalytically active PKA holoenzymes can remain intact within the cytoplasm [138], demonstrating that the compartmentation of cAMP is not yet fully understood.

In conclusion, all data support the existence of subcellular cyclic nucleotide compartments in vascular SMCs. Furthermore, it seems clear that PDEs play a vital role in cAMP compartmentation, emphasizing in particular the role of PDE4. The compartmentation of cAMP involves coupling specific AC to GPCR isoforms that synthesize cAMP at different sites in the cell and bind to other effectors that together form signalosomes. AKAPs that interact with GPCRs, AC, PKA, PDE, and probably other signaling proteins may also act in this process.

### 6.2. Compartmentation of cGMP Signalling

cGMP signalling is involved in cell proliferation, differentiation and contractility of vascular SMCs [5,9,19,125,238]. Therefore, it is imperative to understand the compartmentation of cGMP in vascular smooth muscle. Studies have shown that the increase in cGMP induced by NP and NO donors can occur in different subcellular compartments [5,19,239,240,241]. In 2006, a study by Piggott et al. demonstrated compartmentation of cGMP at the vascular level, and that PDE is not solely responsible for this phenomenon. These authors reported that the functional compartmentation of cGMP signals might underlie the unique actions of ANP and NO since the relative increase in cGMP with NO-sGC stimulation exceeded that with NP-pGC [242]. In 2007, Cawley et al. in rat aorta SMCs observed that NO donors caused transient increases in the concentration of GMP, which depend on the activity of sGC and PDE5 [243]. Nausch et al. (2008), using the cGMP-biosensor FincGs, also observed the existence of subcellular cGMP compartments in rat aorta SMCs; they suggested that PDE5 differentially regulates the effects of NO and NP donors [244]. Other authors reported that PDE3 hydrolyses both cAMP and cGMP. However, the affinity is higher for cGMP than cAMP hydrolysis, which allow cGMP to act as a competitive inhibitor of cAMP hydrolysis [19]. On the other hand, PDE5 is highly specific for cGMP [6,94]. Moreover, Cairrao et al. (2010), using a whole-cell configuration of the patch-clamp technique demonstrated that ANP and testosterone stimulate potassium current (I_K_) in human umbilical artery smooth muscle cells (HUASMCs). This effect corresponds to the activation of BK_Ca_ and K_V_ channels through increased activation of cGMP and PKG. The authors also demonstrated that activation of sGC with NO does not induce a stimulation of the I_K_ current. These results suggest a possible compartmentation underlying the observed effects [204]. Six years later, Feiteiro et al. (2016), using CNG channels as cGMP biosensors in HUASMCs, demonstrated for the first time that cGMP is compartmentalized in human vascular muscle, and controlled by PDE3 and PDE5 [10]. The authors applied a whole-cell configuration of the patch-clamp technique to measure CNG current in infected HUASMCs. The data show differences in the spatiotemporal distribution of intracellular cGMP depending on the activation of two cyclases differently localized: (1) when pGC is activated by ANP, cGMP rises near the membrane; (2) when sGC is activated by NO donors (SNP), cGMP increases in the cytosol and also near the membrane. These data suggest that when a cyclase is activated, the increase of intracellular cGMP is not uniformly distributed within the cell and is probably clustered at specific sites [10].

Furthermore, PDEs play a key role in this compartmentation, because different PDE subtypes (PDE3 and PDE5) regulate particulate and cytosolic cGMP pools. PDE5 appears to control the particulate, but not the soluble pool, and the soluble cGMP pool is under the exclusive control of PDE3. In this sense, our research group demonstrated that PDE5 and PDE3 control the particulate cGMP pool formed near the plasma membrane and the cGMP pool localized in the cytosol is exclusively controlled by PDE3 [10].

In 2017, two research groups showed cGMP compartmentation in the vascular cells. The findings of Wilson et al. (2017) agreed with those observed by Feiteiro et al. It was also demonstrated that the cGMP compartmentation was due to PDE5 and PDE3. This group also revealed the existence of distinct pools of PDE5A in human arterial smooth muscle cells, which they believe can regulate different events dependent on cGMP. Specifically, these authors observed that PDE5A is associated with an IP3R complex that regulates calcium transients and that, in the cytosol, PDE5A can regulate cGMP-mediated inhibition of PDE3A [245]. On the other hand, the Leblais group observed the cGMP compartmentation in cultured RASMCs using the FRET-based cGMP sensor cGi-500. The authors studied the short-and long-term modulation of the cGMP/PDE pathway in controlling vascular cell proliferation. Short-term experiments showed that the basal PDE1 activity had no influence on the intracellular concentration of cAMP but selectively controlled the intracellular concentration of cGMP produced by CNP. PDE1 was activated by Ang II and then became active on the β-adrenoceptor-dependent cAMP pool and the NO-dependent cGMP pool. Thus, the PDE5 controls the concentration of cGMP independently of the nature of the activated GC. The authors also conclude that the PDE9 is an important PDE at the vascular level and regulates the NO-elicited cGMP pool. The long-term experiments show that: (a) PDE1 and PDE9 inhibition induces small but significant reductions in the proliferative effect of FBS in the absence of exogenous cGMP manipulation and (b) PDE5 controls FBS-stimulated proliferation upon cGMP stimuli. In conclusion, the authors show that the cGMP pools from different origins differentially regulate cell proliferation in vascular SMCs [126]. 

**Figure 6:**
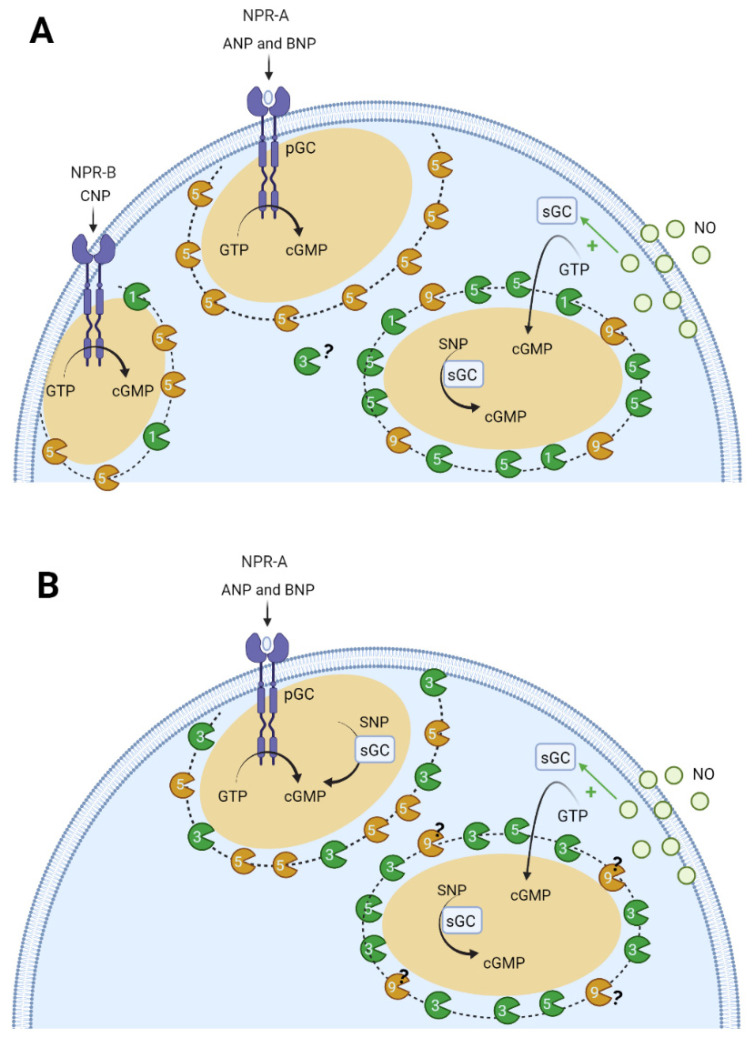
Schematic representation of compartmentation of cGMP signalling in vascular SMCs in (**A**) a synthetic and (**B**) a contractile phenotype (created with biorender.com). In synthetic phenotype (**A**), activating natriuretic peptide receptors A and B leads to a pool of pGC/cGMP mainly controlled by PDE5. Moreover, activation of soluble guanylyl cyclases (sGC) by NO leads to a generation of cGMP in the intracellular medium also mainly controlled by PDE5. In the contractile phenotype (**B**), activating natriuretic peptide receptors A leads to a pool of pGC/cGMP controlled by PDE5 and PDE3. Activation of soluble guanylyl cyclases (sGC) by NO leads to generation of cGMP in the intracellular medium mainly controlled by PDE3.

In conclusion, all data support the existence of subcellular compartments of cyclic nucleotides in vascular SMCs [5,19]. Furthermore, it seems clear that PDEs play an important role in the compartmentation of cGMP, with particular emphasis on PDE1, PDE3, PDE5, and PDE9 [126,238]. cGMP signaling compartmentation seems to exist within two different pools: pGC/cGMP controlled by PDE5 and PDE3, and sGC/cGMP mainly controlled by PDE3 [10]. In addition, the compartmentation of cGMP seems to be regulated by the spatial location of PKG, PKA, and IP3R complex. However, still unclear is the role of other proteins (e.g., myosin, NPR1, and tropomyosin) that can act as PKG scaffolding proteins [232]. So far, the mechanism that causes the formation of these compartments in vascular SMCs continues to be under intense investigation. Figure 6 represents a schematic model of cGMP compartmentation in the vascular smooth muscle cells.

## 7. Compartmentation as a Potential Therapeutic Approach for CVD

Due to the critical role of cyclic nucleotide signalling, cAMP/PKA and cGMP/PKG compartmentation are recognized as essential signalling elements in vascular physiology and pathophysiology. In the context of disease, the complexity of the mechanistic pathways increases [7]. Unfortunately, the details on how cyclic nucleotide compartmentation is compromised are still not fully understood and, therefore, several pharmacological strategies have been suggested.

From a basic to clinical perspective, these strategies involve increasing cAMP production by activating AC [246] or inhibiting PDE [11,217,247,248,249,250] and MRP4 [215], which consequently can activate PKA, EPAC, POPDC and CNG depending on the type of cells [251]. However, this indiscriminate activation can be a problem, causing undesirable effects and therefore limit therapeutic action. Other studies have explored the possibility of developing drugs that target EPAC [252,253,254] to avoid some of these side effects that arise associated with the elevation of the cAMP. For example, specific EPAC 1 agonists help treat vascular inflammation, while EPAC 1 and 2 antagonists appear to be beneficial in the treatment of heart failure [252]. In this sense, the range of concentration of EPAC agonists/antagonists should be chosen to not damage either heart or the smooth muscle vasculature. On the other hand, cGMP production increases by stimulating soluble GC with NO donors (e.g., SNP, diethylamine NONOate, DEA-NO) [255]. However, NO can act independently of cGMP on several target proteins via cysteine S-nitrosylation [256]. Another approach involves the administration of natriuretic peptides as therapeutic designated agents for GC-A/B-cGMP stimulation [257]. Still, these are small peptides that are degraded rapidly by tissue proteases and require continuous intravenous infusion in a hospital setting. To avoid this degradation, activation of NPR with recombinant NP has been developed [54,258]. Furthermore, another novel drug recently synthetized for hypertension is the ANP-analogue (MANP) that induce a decrease of the blood pressure, natriuresis and decrease the production of aldosterone [259,260]. Nesiritide, a recombinant BNP, has been approved for the management of acute HF and to reduce blood pressure in patients with severe uncontrolled hypertension [261]. Furthermore, recent studies have shown that inhibition of ANP and BNP degradation by a neprilysin inhibitor combined with an Ang II receptor blocker improves HF and resistant hypertension in humans [66]. Another approach involves the administration NOS targeting compounds, although its pharmacological efficiency has not yet been demonstrated in clinical trials. The most recoupling NOS are arginine, citrulline and tetrahydrobiopterin, which are indicated for several pathologies such as preeclampsia, peripheral arterial occlusive disease and hypertension [257].

Regarding the sGC stimulators and activators, the main advantage of these drugs is that they do not induce tolerance. Riociguat (BAY 63–2521) was the first approved sGC stimulator for chronic thromboembolic pulmonary hypertension and PAH [262]. The administration of vericiguat (BAY-1021189) and praliciguat is also under investigation. Vericiguat (BAY-1021189) has been approved by Food and Drug Administration (FDA) and entered the U.S. market this year [263]. Ataciguat (HMR1766), cinaciguat (BAY58–2667) and runcaciguat are sGC activators, but due to severe side effects ataciguat’s development was discontinued [264]. The other sGC activators remain under investigation [263].

Neutral endopeptidase (neprilysin, NEP) is a zinc metallopeptidase responsible for the breakdown of many vasoactive mediators, vasoconstrictors (angiotensin II and endothelin) and vasodilators (natriuretic peptides and bradykinin). For this reason, the effect on blood pressure is very small or null. This fact led to the development of new drugs designated vasopeptidase inhibitors, where the effect of NEP inhibitors was associated with angiotensin converting enzyme stimulation [257]. Currently the development of this new class is being explored computationally in order to increase their safety and selectivity for treatment hypertension [265].

On the other hand, the use of PDE inhibitors is one of the most well-established pharmacological strategies to improve CV therapy (for more detailed information, please see this review [266]). In this context, the use of PDE inhibitors as a drug for PAH treatment, intermittent claudication and congestive heart failure (HF) has been approved. Some groups suggest that PDE activation (e.g., PDE4) may be of interest to treat HF [267]. Moreover, PDE3 inhibitors such as cilostazol and milrinone are mostly used for the treatment of HF patients. Cilostazol is approved for intermittent claudication but is associated with several side effects and for this is contraindicated in severe HF patients. Milrinone is approved for severe congestive HF, but its clinical application is very limited, similar to cilostazol, due to its significant side effects [268]. However, the use of PDE3 inhibitors could become clinical practice even for patients with severe HF, i.e., targeting PDE3 or other PDE isoforms specifically in the vasculature without promoting cardiac toxicity. In this sense, the addition of other drugs that increase the levels of cyclic nucleotides, such as MRP4, may be a possible therapeutic approach to produce the same effects without cardiac toxicity. 

PDE5 inhibitors such as sildenafil (marketed as revatio by Pfizer), tadalafil (marketed as Adcirca by Eli Lilly), and vardenafil are presently used for the PAH treatment [11]. Besides these PDE5 inhibitors, sildenafil, vardenafil, tadalafil, and avanafil (Stendra; Vivus), have been highly successful and approved for the treatment of erectile dysfunction. Moreover, dipyridamole, a non-elective PDE inhibitor, inhibits platelet aggregation and is used to prevent post-surgical thromboembolic events and stroke [11]. These PDE5 inhibitors are usually considered safe and well tolerated, and most frequent side effects are headache, flushing, dyspepsia, and vision disturbances [268]. 

Similarly, given cyclic nucleotides’ antiproliferative and antimigratory properties, PDE appears of great interest in these vascular proliferative diseases. In this sense, the properties of PDE3 inhibition, leading to vasodilatation and inhibition of platelet aggregation and vascular SMCs proliferation, appeared to be favourable in atherosclerosis. Therefore, several clinical trials have been designed to evaluate the benefits of the already FDA approved PDE3 inhibitor, cilostazol, for intermittent claudication [269]. In human EC, this PDE inhibitor reduces oxidative stress by increasing NOS phosphorylation in stress-induced premature senescence through up-regulation of sirtuin 1 [270]. Furthermore, Moreira et al. (2018) also demonstrated using rat mesenteric arteries that cilostazol can reverse age-related endothelial dysfunction through mechanisms that promote a reduction in oxidative stress, increased NO bioavailability and EDHF-mediated relaxation [271]. In this sense, we conclude that this drug may soon be a valuable tool for the treatment of atherosclerosis. Moreover, electrophysiological studies (patch-clamp experiments) are an added value allowing the addition of cAMP or cGMP in the patch pipette filling solution, which helps cyclic nucleotide dialysis into the intracellular medium and allows the study of PKA and PKG in more detail. Together, the use of selective inhibitors of the PKA [272] or PKG [273] catalytic site together with the selective cyclic nucleotide analogues, would allow a more detailed study of the signaling pathways. However, this approach also has limitations due to the limited selectivity of cyclic nucleotide analogues and pharmacological modulators. In addition, unexpected effects such as PDE inhibition can be observed [7], making it challenging to interpret the results obtained with these molecules.

In conclusion, understanding cyclic nucleotide signalling is fundamental since the actions of the local cyclic nucleotide (compartmentation) and the interaction of the two signalling pathways are implicated in pathophysiological conditions. In this sense, the use of PDE inhibitors, mainly PDE5 and PDE3, is one of the most well-established pharmacological strategies to improve CV therapy. On the other hand, the EPAC 1 and 2 antagonists and selective inhibitors from the PKA or PKG catalytic site, together with the cyclic nucleotide analogues, are new therapeutic strategies currently in development. cGMP-modulating drugs approved or under clinical investigation for therapeutic vascular applications are NO donors, NOS targeting compounds, soluble GC (GC-1/2) stimulators and activators, GC-A/B stimulators, NEP inhibitors, and PDE inhibitors. However, this work indicates that combining these different pathways will be a crucial therapeutic strategy.

## 8. Conclusions and Future Perspectives

Since discovering cyclic nucleotides, several studies have attempted to understand how their compartmentalized signaling plays a critical role in cardiovascular pathophysiology. Understanding the interactions between the different cyclic nucleotide effectors and the crosstalk between the two signalling pathways provides insight into the compartmentation of cyclic nucleotides in vascular smooth muscle. In this sense, some innovative techniques have been an asset to demonstrate protein-protein interactions, improving the vascular research in this field. Furthermore, a deeper understanding of these mechanisms clarifies the most relevant axes for the regulation of vascular tone and allows the detection of possible changes associated with pathological disorders useful in the discovery of new drugs. In the future, it is suggested that subsequent laboratory studies be carried out to clarify the role of PDE3 in the compartmentation of SMCs in the synthetic phenotype, as well as PDE1 and PDE9 in the compartmentation of SMCs in the contractile phenotype. From a clinical perspective, we suggest evaluating the use of other PDE inhibitors (in addition to those already established for PDE5 and PDE3) to improve CV therapy. Moreover, another pharmacological strategy involving EPAC 1 and 2 antagonists, selective inhibitors of PKA or PKG, NOS-directed compounds, soluble GC stimulators and activators (GC-1/2), GC-A/B stimulators and NEP inhibitors, should be performed.

## Figures and Tables

**Table 1:** PDEs expressed in vascular smooth muscle and their expression according to SMCs phenotype.

Cyclic Nucleotide Phosphodiesterases (PDEs)	Selectivity for cAMP Hydrolysis	Selectivity for cGMP Hydrolysis	Dual Selectivity Hydrolysis	Synthetic Phenotype	Contractile Phenotype
PDE1A		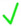		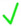	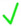
PDE1B		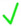			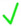
PDE1C			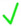	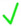	
PDE3A			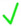	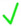	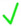 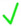
PDE3B				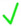	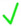
PDE4D	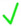			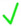 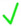 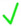	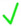 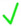 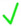
PDE5A		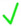		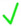 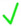 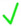	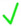 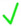 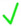
PDE7A	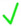			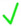	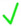
PDE7B				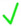	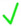 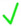
PDE8A	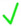			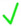 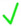	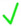
PDE9		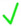		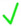	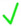
PDE10A			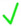	?	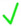

## Abbreviations

AC—adenylyl cyclases; AKAP—A-kinase anchoring proteins; Ang II—angiotensin II; ANP—atrial natriuretic peptide; ATP—adenosine triphosphate; BNP—brain natriuretic peptide; Ca^2+^—calcium; CaM—calmodulin; CaMKII—Ca^2+^/calmodulin-dependent protein kinase II; cAMP —cyclic adenosine-3′,5′-monophosphate; cGMP—cyclic guanosine-3′,5′-monophosphate; cIMP—inosine 3′,5′-cyclic monophosphate; CNG—cyclic nucleotide-gated; CO—carbon monoxide; cryo-EM—cryo-electron microscopy; CV—cardiovascular; CVD—cardiovascular diseases; EC—endothelial cells; eNOS—endothelial nitric oxide synthase; EPAC—exchange protein directly activated by cAMP; FRET—fluorescent energy transfer resonance; FSK—forskolin; GC—guanylyl cyclases; GPCR—G-protein-coupled receptors; Gs—stimulatory G protein; GTP—guanosine triphosphate; iNOS—inducible NOS; KHD—kinase homology domain; L-Type VOCC—L-type voltage-operated Ca^2+^ channels; MLC—myosin light chain; MLCK—myosin light chain kinase; nNOs—neuronal nitric oxide synthase; NO—nitric oxide; NOS—nitric oxide synthase; NP—natriuretic peptides; NP—natriuretic peptides; PAH—pulmonary arterial hypertension; PDEs—cyclic nucleotide phosphodiesterases; pGC—particulate GC; PH—pulmonary hypertension; PKA—protein kinase A; PKC—protein kinase C; POPDC—Popeye domain-containing proteins; PPi—pyrophosphate; RASMCs—rat aortic smooth muscle cells; ROCK—Rho kinase; sGC—soluble GC; SICM—scanning ion conductance microscopy; SMCs—smooth muscle cells; SR—sarcoplasmic reticulum.
